# Comparative Evolutionary Histories of the Fungal Chitinase Gene Family Reveal Non-Random Size Expansions and Contractions due to Adaptive Natural Selection

**DOI:** 10.4137/ebo.s604

**Published:** 2008-03-18

**Authors:** Magnus Karlsson, Jan Stenlid

**Affiliations:** Department of Forest Mycology and Pathology, Swedish University of Agricultural Sciences, P.O. 7026, SE-75007, Uppsala, Sweden

**Keywords:** chitinases, gene family, fungi, evolution, phylogeny

## Abstract

Gene duplication and loss play an important role in the evolution of novel functions and for shaping an organism’s gene content. Recently, it was suggested that stress-related genes frequently are exposed to duplications and losses, while growth-related genes show selection against change in copy number. The fungal chitinase gene family constitutes an interesting case study of gene duplication and loss, as their biological roles include growth and development as well as more stress-responsive functions. We used genome sequence data to analyze the size of the chitinase gene family in different fungal taxa, which range from 1 in *Batrachochytrium dendrobatidis* and *Schizosaccharomyces pombe* to 20 in *Hypocrea jecorina* and *Emericella nidulans*, and to infer their phylogenetic relationships. Novel chitinase subgroups are identified and their phylogenetic relationships with previously known chitinases are discussed. We also employ a stochastic birth and death model to show that the fungal chitinase gene family indeed evolves non-randomly, and we identify six fungal lineages where larger-than-expected expansions (Pezizomycotina, *H. jecorina*, *Gibberella zeae*, *Uncinocarpus reesii*, *E. nidulans* and *Rhizopus oryzae*), and two contractions (*Coccidioides immitis* and *S. pombe*) potentially indicate the action of adaptive natural selection. The results indicate that antagonistic fungal-fungal interactions are an important process for soil borne ascomycetes, but not for fungal species that are pathogenic in humans. Unicellular growth is correlated with a reduction of chitinase gene copy numbers which emphasizes the requirement of the combined action of several chitinases for filamentous growth.

## Introduction

Chitin is a polymer which consists of N-acetylglucosamine monomers (GlcNAc), linked by β-1,4-glucosidic bonds. It is widely distributed in nature and it is a constituent of the exoskeleton of invertebrates, of zooplankton and of fungal cell walls. Chitinases (EC 3.2.1.14) hydrolyze the bonds between GlcNAc residues releasing oligomeric, dimeric (chitobiose) or monomeric (GlcNAc) products. Chitobiose can be further cleaved by N-acetylhexosaminidases (EC 3.2.1.52) into GlcNAc (Keyhani and Roseman, 1999). Chitinases are divided into two different glycoside hydrolase families (18 and 19) based on amino acid sequence similarity (Henrissat, 1991; Henrissat and Bairoch, 1993). These two families share limited similarity at the amino acid level and have different three-dimensional structures and modes of action (Iseli et al. 1996). These enzymes can display either exo- or endoactivity, depending on the structure of the catalytic site (Terwisscha van Scheltinga et al. 1994; van Aalten et al. 2000; van Aalten et al. 2001).

Growth and morphological development of fungi makes cell wall remodelling a necessity. Cell expansion and division, spore germination, hyphal branching and septum formation all depend on the activities of hydrolytic enzymes intimately associated with the fungal cell wall, among them chitinases (Adams, 2004). Chitinases are also implied in autolysis and recycling of older parts of the fungal mycelia (Duo-Chuan, 2006). Chitinases also have aggressive roles as fungal pathogenicity factors during infection of other fungi (mycoparasitism), insects and nematodes (Wattanalai et al. 2004; Duo-Chuan, 2006; Gan et al. 2007a). Furthermore, chitinases are involved in degradation of chitin for nutritional needs (Duo-Chuan, 2006). Lysis of the host cell wall and degradation of nematode egg shells are shown to be important steps in the mycoparasitic and nematophagous attack (Howell, 2003; Benitez et al. 2004; Gan et al. 2007a), and hence chitinases from various fungi used as biocontrol agents have been cloned and characterised (Felse and Panda, 1999; Hoell et al. 2005; Klemsdal et al. 2006; Gan et al. 2007a; Gan et al. 2007b; Dong et al. 2007).

The diversity of chitinase function during the fungal life cycle raises interesting questions regarding the evolution of this important gene family. Fungal chitinases belong to glycoside hydrolase family 18 (GH18) and they consist of discrete domains, which are variously arranged in different orders in different proteins (Gilkes et al. 1991; Warren, 1996; Henrissat and Davies, 2000). Besides the catalytic domain there is very often a substrate-binding domain present. These substrate-binding domains are not necessary for chitinolytic activity, although they seem to enhance the efficiency of the enzymes (Suzuki et al. 1999; Limon et al. 2001).

There is a large variation in the number of GH18 genes present in different fungal genomes, from 1 in *Schizosaccharomyces pombe* to 20 in *Hypocrea jecorina* (Seidl et al. 2005). This implies that the size of the fungal GH18 gene family has been highly dynamic throughout evolution. Gene duplication is an important process that can contribute to the evolution of novel functions. However, the mechanisms that govern the fate of duplicated genes are not very well understood. Recent progress suggests that stress-related genes frequently are exposed to duplications and losses, while growth-related genes show selection against change in copy number (Wapinski et al. 2007). High copy-numbers of stress-responsive genes may be beneficial by allowing adaptations to diverse ecological niches. Recent paralogues diversify most frequently at the level of regulation, and more rarely at the level of biochemical function (Wapinski et al. 2007).

Identification of expansions as well as contractions of protein families in fungi with diverse ecological roles can aid in understanding relationships between function and phylogeny. The fungal GH18 gene family constitutes an interesting case study of gene duplication and loss, as their biological roles includes growth and development as well as more stress-responsive functions. Hence it is possible to test the hypothesis that growth-related genes display selection against changes in copy number while stress-related genes tolerate more duplications and losses, within a single gene family. Here we present a study where we use genome sequence data to analyze the size of the GH18 gene family in different fungal taxa and to infer their phylogenetic relationships. We also employ a stochastic birth and death model to test for non-random evolution of the fungal GH18 gene family. We show that the fungal GH18 gene family indeed evolves non-randomly and we identify fungal lineages where larger-than-expected expansions or contractions potentially indicate the action of adaptive natural selection.

## Materials and Methods

### Biomining of genome sequences

In order to avoid sampling bias our study was restricted to fungal species where genome sequence information and estimates of divergence times were available. *In silico* translated gene products from individual fungal genome sequences were screened for the presence of GH18s using BLASTP (Altschul et al. 1997) in an iterative process. Fungal genomes were available at the homepages of the DOE Joint Genome Institute (http://www.jgi.doe.gov/), the Fungal Genome Initiative at the BROAD Institute (http://www.broad.mit.edu/annotation/fgi/) or at Génolevures (http://cbi.labri.u-bordeaux.fr/Genolevures/blast/index.php). The 18 published GH18s from *H. jecorina*, Chi18-1 through Chi18-18, and two additional proteins annotated in the genome, Chi18-rel1 (Chi18-20) and Chi18-rel2 (Chi18-19), protein ID 65162 and 121355 in version 2.0 were used as starting material, as this is so far the highest number of GH18s from a single fungal species (Seidl et al. 2005). Later on the number of proteins used was reduced to Chi18-1, Chi18-2, Chi18-12 and Chi18-19 from *H. jecorina* as these representatives provided the same information as the larger set. In addition, the GH18s that were identified by the first round of similarity searches in a target genome was iteratively used in a second round of BLAST searches against the same genome. The protein identifiers from the respective genome sequencing projects were used during subsequent analyses, except for when the protein was characterized and named.

### Phylogenetic analysis

Amino acid sequences of GH18 catalytic domains were determined by InterProScan (Zdobnov and Apweiler, 2001) or Conserved Domain Database searches (Marchler-Bauer et al. 2005). Sequences were manually trimmed and aligned with Clustal X (Thompson et al. 1997) and inspected using BioEdit (Hall, 1999). Amino-acid similarity between sequences was calculated using MegAlign, implemented in the DNASTAR program package (DNASTAR, Madison, WI). Phylogenetic analysis of catalytic domains was performed using maximum likelihood methods implemented in PhyML-aLRT 1.1 (Guindon and Gascuel, 2003; Anisimova and Gascuel, 2006). The JTT amino-acid substitution model (Jones et al. 1992) was used, the proportion of invariable sites was set to 0, one category of substitution rate was used and gaps were treated as unknown characters. The starting tree to be refined by the maximum likelihood algorithm was a distance-based BIONJ tree estimated by the program (Guindon and Gascuel, 2003). Statistical support for phylogenetic grouping was assessed by approximate likelihood-ratio tests based on a Shimodaira-Hasegawa-like procedure (SH-aLRT) (Anisimova and Gascuel, 2006) and by bootstrap analysis (500 resamplings).

### Likelihood analysis of gene gain and loss

In order to statistically test whether the size of the fungal GH18 gene family is compatible with a stochastic birth and death model we used the program CAFE (Computational Analysis of gene Family Evolution) (De Bie et al. 2006), which is based on the probabilistic framework developed by Hahn et al. (2005). From a specified phylogenetic tree and the gene family size in extant species, we inferred the most likely gene family size at internal nodes, tested for accelerated rates of gene family expansions or contractions and identified the branches that are responsible for the non-random evolution.

**Figure 1:**
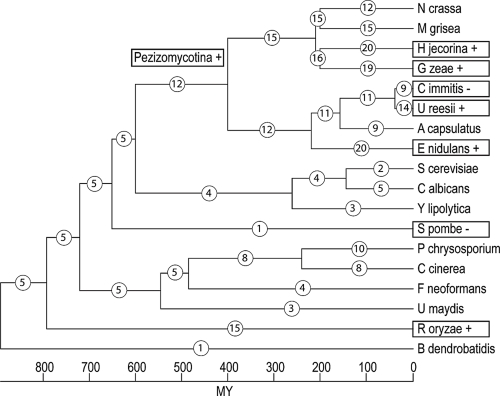
Distribution of GH18 gain and loss among fungal lineages. Phylogenetic relationships among the fungal species used in the current study are shown, including divergence dates in millions of years (Taylor and Berbee, 2006). Circled numbers represent total number of GH18 genes in extant species and estimates of total number of GH18 genes for ancestral species. Boxed taxon names indicates a significant (*p*-values ≤ 0.05 or Likelihood ratios >50) expansion (+), or a significant contraction (−) of the GH18 gene family size.

The fungal GH18 gene family data in extant species that were used in the current analysis are found in Table 1. Fungal GH18s can be divided into three major phylogenetic clusters, A, B and C, and further subdivisions within these clusters are made (Seidl et al. 2005). Our phylogenetic analysis shows that cluster C GH18s can be merged with cluster A and therefore we analysed the data in three ways; cluster A GH18s separately, cluster B GH18s separately and all GH18s merged. CAFE assumes that the gene family under study is present in the most recent common ancestor of all taxa included in the analysis. Therefore *Batrachochytrium dendrobatidis* was excluded from the analysis of cluster B GH18s. The phylogenetic relationships between the species that were included in the analysis are shown in Figure 1, with branch lengths in millions of years. Phylogenetic relationships and estimations of divergence times were taken from previous publications (Bowman et al. 1996; Kasuga et al. 2002; Padovan et al. 2005; Taylor and Berbee, 2006), assuming that the Devonian ascomycete *Paleopyrenomycites devonicus* (Taylor et al. 2005) represents Pezizomycotina (Taylor and Berbee, 2006) which gives an estimated age of 923 millions of years for the fungal phylum.

Alternative estimates of divergence times can be made by assuming that *P. devonicus* represents Sordariomycetes (estimated age of the fungal phylum at 1630 millions of years) or Ascomycota (estimated age of the fungal phylum at 495 millions of years) as outlined in Taylor and Berbee, (2006), although these alternative estimates resulted in more improbable age estimates when compared with age estimates in other phyla. However, these alternative estimates were included in the analysis although *Coccidioides immitis*, *Uncinocarpus reesii*, *Ajellomyces capsulatus* and *B. dendrobatidis* were excluded because of incompatibility of divergence estimates (Bowman et al. 1996; Kasuga et al. 2002; Padovan et al. 2005; Taylor and Berbee, 2006). The four different phylogenetic trees used in the current study including branch lengths are found in Supplemental information (S1).

The birth and death parameter (λ) was estimated from the data (De Bie et al. 2006) and was 0.001 for all datasets. *p*-values were computed using 1000 re-samplings and identification of the branch that was the most likely cause of deviations from a random model was determined by Viterbi, Branch-cutting and Likelihood ratio test procedures (De Bie et al. 2006). We considered *p*-values ≤ 0.05 or likelihood ratios above 50 to be significant for branch identification.

## Results

**Figure 2:**
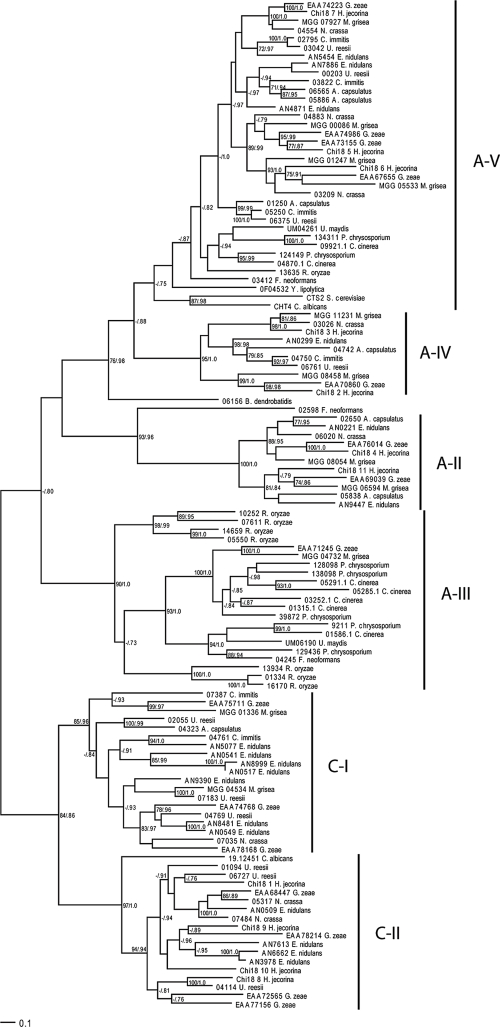
Phylogenetic relationships of fungal cluster A family 18 glycoside hydrolase catalytic domains. Phylogenetic analyses were performed using maximum likelihood methods as implemented in PhyML-aLRT, based on an alignment of family 18 glycoside hydrolase catalytic domain amino acid sequences. Branch support values (bootstrap proportions/approximate likelihood-ratio test probabilities) are associated with nodes, with a dash indicating that the support was <70%/0.70. The bar marker indicates the number of amino-acid substitutions. Protein identifiers include protein name, GenBank accession nos. or locus/protein ID from the respective genome projects. Group names are indicated, see text for reference. Cluster B GH18 Chi18-12 from *H. jecorina* was used as outgroup (not shown).

**Figure 3:**
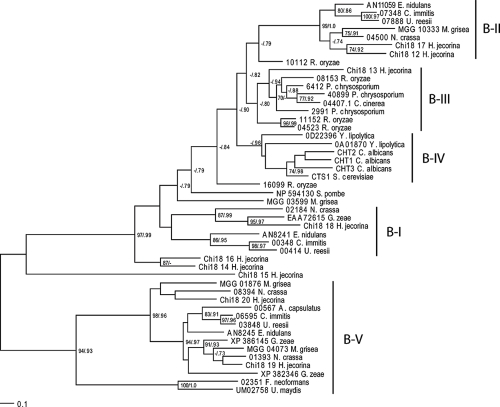
Phylogenetic relationships of fungal cluster B family 18 glycoside hydrolase catalytic domains. Phylogenetic analyses were performed using maximum likelihood methods as implemented in PhyML-aLRT, based on an alignment of family 18 glycoside hydrolase catalytic domain amino acid sequences. Branch support values (bootstrap proportions/approximate likelihood-ratio test probabilities) are associated with nodes, with a dash indicating that the support was <70%/0.70. The bar marker indicates the number of amino-acid substitutions. Protein identifiers include protein name, GenBank accession nos. or locus/protein ID from the respective genome projects. Group names are indicated, see text for reference. Cluster A GH18 06156 from *B. dendrobatidis* was used as outgroup (not shown).

There was considerable variation in the number of GH18 genes between different fungal species, ranging from 1 in *B. dendrobatidis* and *S. pombe* to 20 in *H. jecorina* and *Emericella nidulans* (Table 1). Because of the large variation in length and domain structure of fungal GH18s (Seidl et al. 2005), only the GH18 catalytic domains were used for phylogenetic analysis. Fungal GH18s were divided into two major clusters, A and B (Figs. 2 and 3), each subdivided into groups. Cluster A sequences formed six separate groups that were equivalent with the A-II, A-III, A-IV, A-V, C-I and C-II groups described by Seidl et al. (2005) (Fig. 2). Cluster B sequences formed five separate groups, here referred to as B-I through B-V (Fig. 3). Groups B-I and B-II were described previously (Seidl et al. 2005). The average number of cluster A GH18 genes in the included species was 6.6, ranging from 0 (*S. pombe*) and 17 (*E. nidulans*). This average was higher than for cluster B GH18 genes where the average was 2.8 genes per species, ranging from 0 (*B. dendrobatidis*) and 9 (*H. jecorina*). The different GH18 subgroups were characterized by considerable differences in levels of amino-acid conservation. Subgroups A-II, A-IV, A-V and B-V showed the highest levels of mean interspecific amino-acid identity between species in Eurotiales (Table 2). Members in subgroups C-I and C-II displayed the lowest levels of conservation while levels in B-I and B-II were intermediate to the other groups (Table 2).

To investigate the evolutionary change of the number of GH18 genes in fungi, we estimated the number of genes in ancestral species and the number of gene gains and losses for each branch of the phylogenetic tree of the fungal species (Fig. 1). The analysis showed that the fungal GH18 gene family, analysing both cluster A and B together, as well as cluster A GH18s alone have evolved non-randomly (p < 0.001, Table 3). Six branches were identified as contributing to this non-random pattern, including 5 expansions and 1 contraction (Table 3). These branches included the ancestor to the Pezizomycotina clade, as well as extant species *C. immitis*, *U. reesii*, *E. nidulans*, *Gibberella zeae* and *Rhizopus oryzae*. Analysis of gene phylogenies of GH18 subgroups, identified subgroups C-I and C-II as the likely targets responsible for the non-random expansion seen in *E. nidulans* and *U. reesii* (Fig. 2). The contraction in *C. immitis* probably took place in subgroup C-II (Fig. 2). It was not possible to identify the target subgroup in *G. zeae*, although C-I and C-II appeared to have expanded compared to other Sordariomycetes (Fig. 2, Seidl et al. 2005). It should be noted though, that an additional gene was also present in subgroups A-III and A-V, as compared with the closely related *H. jecorina* (Fig. 2, Seidl et al. 2005). The expansion seen in cluster A GH18s in *R. oryzae* took place in subgroup A-III (Fig. 2). Although cluster B GH18s did not display a non-random pattern of evolution as a whole (p = 0.568), two branches were still identified where significant expansions took place (Table 3). For both *H. jecorina* and *R. oryzae* this expansion took place in the large B-I/II/III/IV cluster (Fig. 3). Analyzing all GH18s together using *R. oryzae* as the most recent common ancestor of all taxa instead of *B. dendrobatidis* resulted in a significant contraction in *S. pombe* (Table S2).

Taylor and Berbee, (2006) also published two alternative estimates of divergence times of fungal taxa, although these alternative estimates resulted in more improbable age estimates when compared with age estimates in other phyla. Analyzing the evolution of the GH18 gene family using these alternative estimates was performed to assess the robustness of the analysis to differences in divergence dates. The two more recent estimates of divergence dates (estimated age of the fungal phylum at 495 or 923 millions of years) both showed that the GH18 family have evolved non-randomly (p < 0.001, Table S2), and identified the same branches as contributing to this non-random pattern (Table S2). The oldest estimate (estimated age of the fungal phylum at 1630 millions of years) gave no significant changes in size of the GH18 gene family (Table S2).

**Figure 4:**
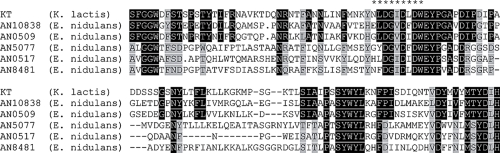
Partial alignment of *K. lactis* zymocin with subgroups C-I and C-II members in *E. nidulans*. Identical residues in a column are indicated in white and boxed in black, two different residues in a column are indicated by gray boxes, gaps are indicated by dashes. The conserved GH18 gene family active site residues are indicated by asterisks. AN10838 and AN0509 represents the C-II GH18 subgroup, AN5077, AN0517 and AN8481 represents the C-I subgroup, KT = Killer Toxin α-subunit.

The involvement of GH18 subgroups C-I and C-II in both expansions and contractions in ascomycetes qualified them for further study. Based on the genome sequence, C-I members in *E. nidulans* were characterised by Chitin-binding type 1 domains (InterPro acc. no. IPR001002), while C-II members in *E. nidulans* contained LysM peptidoglycan binding domains (InterPro acc. no. IPR002482) in addition to Chitin-binding type 1 domains. These proteins showed considerable similarity with the α-subunit of the yeast killer toxin from *Kluyveromyces lactis* (Fig. 4). The A-III members in *R. oryzae* were short and contained no other domains than the GH18 catalytic domain. The domain-structure of cluster B GH18s in *H. jecorina* has been described before (Seidl et al. 2005) and were characterized by fungal cellulose binding domains (InterPro acc. no. IPR000254). The domain-structure of *R. oryzae* cluster B members included both carbohydrate-binding family V/XII domains (InterProacc. no. IPR003610) and GH18 carbohydrate binding domains (InterPro acc. no. IPR005089).

## Discussion

Accurate estimates of divergence times for fungi are notoriously hard to obtain due to a very limited fossil record. In the current study we have used three different time estimates on our GH18 gene family data. The differences relates to whether the Devonian ascomycete *P. devonicus* (Taylor et al. 2005) represents Sordariomycetes, Pezizomycotina or Ascomycota (Taylor and Berbee, 2006). Using the Sordariomycete calibration result in no significant changes in the GH18 gene family due to the estimated ancient origin of the fungal phylum (1630 millions of years), more than three times the age of the first fossil evidence of land plants (Taylor and Berbee, 2006). The results when using the two divergence estimates based on *P. devonicus* as a representative for Pezizomycotina (origin of fungal phylum at 923 millions of years) or Ascomycota (origin of fungal phylum at 495 millions of years) are very similar, which show a certain level of robustness of the analysis, even for large differences in divergence estimates. Furthermore, the Pezizomycotina estimate has been shown to result in least number of improbable time estimates when compared with other phyla (Taylor and Berbee, 2006).

The number of GH18 genes in different fungal species varies considerably. An expansion in size of a particular gene family or subgroup within a gene family, such as GH18s, suggests that this gene family or subgroup has been important for the fitness of the species during evolution. The observed variation could possibly be attributed to differences in morphology, growth patterns, nutrient acquisition or antagonistic ability between species. Our approach can be used to establish links between phylogeny of the GH18 gene family with the ecological role of the species and to identify specific subgroups as important evolutionary targets in specific fungal lineages.

Filamentous ascomycetes generally possess larger number of GH18 genes as compared with other fungal groups. This larger GH18 gene family size can possibly be attributed to a larger gene copy-number in certain subgroups, but more importantly to the presence of several GH18 subgroups that appear to be unique for filamentous ascomycetes (A-II, C-I and C-II). Subgroups C-I and C-II are identified as the most likely target for the observed expansion in *E. nidulans*, *U. reesii*, *G. zeae* and the ancestor to Pezizomycotina. These GH18 genes share extensive homology with the α-subunit of the yeast killer toxin from *K. lactis*. This yeast killer toxin, zymocin, consists of three subunits (α, β and γ) where toxicity relies solely on the γ-subunit and the α- and β-subunits function in the delivery of γ inside the cell by permeabilization of the yeast cell wall and membrane (Stark et al. 1990; Magliani et al. 1997). This has led to the suggestion that the C-I and C-II GH18s are involved in a similar mechanism in aggressive fungal-fungal interactions, by permeabilization of the cell wall and membrane to enable penetration of antifungal molecules into the antagonist (Seidl et al. 2005). This role is supported by expression data; the C-II member *chi18-10* from *H. atroviridis* is only expressed during growth on fungal cell walls and during plate confrontation assays, but not by carbon starvation or chitin exposure (Seidl et al. 2005). The expansion of C-I and C-II GH18s suggests that interspecific interactions are an important process for soil borne ascomycetes. It also supports the idea that genes involved in stress-related functions can tolerate, or are even under selection for, increases in copy number (Wapinski et al. 2007).

Another intriguing result is the different evolutionary trajectories of the GH18 gene family between the human pathogen *C. immitis* and the closely related *U. reesii*. An expansion of GH18 subgroup C-II in saprotrophic *U. reesii* is in contrast to a contraction in the same subgroup in the pathogenic *C. immitis*, although these species are very closely related (Bowman et al. 1996; Kasuga et al. 2002). This difference should be related to the different life-styles of the two fungi, and we hypothesize that the expansion of C-II seen in *U. reesii* is a consequence of the need for antagonistic interactions with other soil-dwelling fungi. The contraction in *C. immitis* may be reflecting adaptation to a pathogenic lifestyle, where antagonistic interactions with other fungi are minimized. Another human pathogen, *A. capsulatus*, also contains the same number of GH18 genes (9) as *C. immitis* and also lacks subgroup C-II members completely, which indicate that the function performed by C-II members of the GH18 protein family is dispensable for the human pathogenic lifestyle.

The high interspecific sequence variability in C-I and C-II as compared with other GH18 subgroups can be interpreted as the result of diversifying selection. Diversifying evolution due to positive selection is reported for plant chitinases that function as defence proteins against invading fungal pathogens (Bishop et al. 2000; Tiffin, 2004) and in reproductive proteins, in animals and plants (Clark et al. 2006) as well as in fungi (Karlsson et al. 2008). It is possible that the high sequence variability in C-I and C-II represents an adaptation towards differences in cell wall composition in antagonistic species. The significant expansion of the fungal GH18 gene family in the ancestor of the Pezizomycotina probably reflects the emergence of the unique C-I and C-II GH18s in filamentous ascomycete fungi, although the ecological factors driving the selection for these subgroups remain obscure. Although very speculative, the emergence of C-I and C-II GH18s coincide with the estimated time of colonization of terrestrial environments by plants (Sanderson, 2003), which suggests the possibility that the emergence of terrestrial plants created new ecological niches where filamentous ascomycetes could expand into to compete for space and nutrients.

Another expansion took place in cluster B GH18s in *H. jecorina*, which is closely related to mycoparasitic fungi such as *H. lixii*, *H. virens* and *H. atroviridis*. Seidl et al. (2005) reported that certain cluster B GH18 genes from *H. jecorina* have high similarity to GH18s from entomopathogens, such as *Metarhizium anisopliae*, which suggests an aggressive role of these proteins in chitin degradation. Again, there is expression data that support this role; *chi18-13* from *H. atroviridis* is up-regulated during growth on fungal cell walls and during plate confrontation assays (Seidl et al. 2005). The fact that two GH18 subgroups that are implied in aggressive fungal-fungal interactions have expanded significantly during fungal evolution suggests that interspecific antagonistic interactions are important determinants of fungal evolution, community development and functioning. This result is in line with the idea that genes involved in stress-related functions can tolerate, or are even under selection for, increases in copy number (Wapinski et al. 2007).

Species with yeast-like or monocentric growth styles such as *Saccharomyces cerevisiae*, *Yarrowia lipolytica*, *S. pombe* and *B. dendrobatidis* all have low numbers of GH18 genes (2, 3, 1 and 1 genes) as compared with other fungi, even with closely related species exhibiting filamentous growth e.g. *Candida albicans* (5 genes). This suggests that filamentous growth requires the combined action of several GH18s as compared with a yeast-like growth style, even though the reduction in the non-filamentous species are only significant for *S. pombe* (p = 0.023).

The analysis of the GH18 gene family size in basidiomycetes shows no conflict with a random process. However, 5 of the 8 GH18 genes in *Coprinopsis cinerea* and 5 of the 10 GH18 genes in *Phanerochaete chrysosporium* are found in subgroup A-III, compared to only one representative from *Ustilago maydis* and *Filobasidiella neoformans*. Further studies of more closely related species with shorter coalescence times will be needed to determine if the apparent expansion of GH18 subgroup A-III in saprotrophic basidiomycetes and the apparent contraction in pathogenic basidiomycetes is a consequence of the different life-styles of the species. Based on the observed expansion of GH18 subgroups involved in interspecific interactions in ascomycetes, we can hypothesize about the involvement of A-III GH18s in fungal-fungal interactions in basidiomycetes.

Our analysis shows the usefulness of the combination of a stochastic birth and death model and phylogenetic information in a probabilistic framework for identification of lineages with unusually evolving gene families. However, the birth and death model assumes independence among individual genes. This means that any large-scale chromosome duplication, deletion or polyploidization that acts on several gene family members at once violates the assumption of the model (Hahn et al. 2005). Interpretation of gene family size differences between taxa that are separated by genome duplications should be made with caution. There are indications of a recent polyploidization in *R. oryzae* (Taylor and Berbee, 2006) which suggest the possibility that the observed non-random GH18 family size in this species may not be entirely related to adaptive selection. On the other hand, the two GH18 genes in the duplicated *S. cerevisiae* genome (Kellis et al. 2004) belong to the fundamentally different A and B clusters. This indicate that the loss of one of the duplicated *CTS1* and *CTS2* paralogues have been selected for during evolution after whole-genome duplication. Cts1 and cts2 are involved in cell separation during budding and in sporulation (Kuranda and Robbins, 1991; Giaever et al. 2002), which is in line with the idea that genes involved in growth-related functions are under selection against changes in copy numbers (Wapinski et al. 2007).

Another violation against the assumption of independence among individual genes may be seen in *Neurospora crassa*. This species has the lowest number of GH18 genes (12) among the Sordariomycetes, which may be attributed to the presence of a wide array of genome defence mechanisms, including repeat-induced point mutations, greatly slowing down the creation of new genes (Galagan et al. 2003). The low number of GH18 genes in this species is not significantly violating a random process (p = 0.256), but interpretation of data for other gene families in *N. crassa* should be done with caution.

In the current study we have used fungal genome data in comparative work to infer phylogenetic relationships in the fungal GH18 gene family and to detect non-random expansions and contractions. This approach can be used to establish links between phylogeny of the GH18 gene family with the ecological role of the species, and identify specific GH18 subgroups as important evolutionary targets in specific fungal lineages. Within the fungal GH18 gene family we observe selection against changes in copy number in GH18s involved in growth and development as well as selection for increased copy number in GH18s involved in stress-related functions, supporting the idea of a bipolar principle that governs tolerance to duplications and losses (Wapinski et al. 2007). The results also indicate that antagonistic fungal-fungal interactions constitute an important evolutionary force in soil borne ascomycetes, but not for fungal species that are pathogenic in humans.

## Supplemental Information

Tree File S1. Phylogenetic trees with estimates of divergence times used in the current study. **Tree 1.** Phylogenetic tree and estimates of divergence time in millions of years for all included fungi based on the Pezizomycotina calibration.
((((((((N_crassa:200, M_grisea:200):10,(H_jecorina:200, G_zeae:200):10):190,(((C_immitis:38, U_ reesii:38):119, A_capsulatum:157):63, E_nidulans:220):180):200,((S_cerevisiae:145, C_ albicans:145):115, Y_lipolytica:260):340):50, S_pombe:650):70,(((P_chrysosporium:240, C_cinerea:240):245, F_ neoformans:485):60, U_maydis:545):175):72, R_oryzae:792):101, B_dendrobatidis:893);
**Tree 2.** Phylogenetic tree and estimates of divergence time in millions of years for a subset of the included fungi based on the Ascomycota calibration.
(((((((N_crassa:120, M_grisea:120):10,(H_jecorina:120, G_zeae:120):10):90,(C_ immitis:130, E_nidul ans:130):180):90,((S_cerevisiae:90, C_albicans:90):180, Y_lipolytica:270):90):140, S_ pombe:390):60,(((P_chrysosporium:150, C_cinerea:150):150, F_neoformans:300):40, U_ maydis:340):110):30, R_oryzae:480);
**Tree 3.** Phylogenetic tree a nd estimates of divergence time in millions of years for a subset of the included fungi based on the Pezizomycotina calibration.
(((((((N_crassa:200, M_grisea:200):10,(H_jecorina:200, G_zeae:200):10):190,(C_ immitis:220, E_nidu lans:220):180):200,((S_cerevisiae:145, C_albicans:145):115, Y_lipolytica:260):340):50, S_ pombe:650):70,(((P_chrysosporium:240, C_cinerea:240):245, F_ neoformans:485):60, U_ maydis:545):175):72, R_oryzae:792);
**Tree 4.** Phylogenetic tree and estimates of divergence time in millions of years for a subset of the included fungi based on the Sordariomycete calibration.
(((((((N_crassa:400, M_grisea:400):10,(H_jecorina:400, G_zeae:400):10):290,(C_ immitis:420, E_nidu lans:420):280):480,((S_cerevisiae:280, C_albicans:280):590, Y_lipolytica:870):310):130, S_ pombe:1310):160,(((P_chrysosporium:500, C_cinerea:500):480, F_ neoformans:980):120, U_ maydis:1100):370):130, R_oryzae:1600);

**Table S2:** Non-randomly evolving branches in the fungal GH18 gene family using alternative calibration points for dating fungal divergences.

Data set	Branch ID	Sordariomycetes ^1^ *p*-value ^4^	Likelihood ratio ^4^	Pezizomycotina ^2^ *p*-value ^4^	Likelihood ratio ^4^	Ascomycota ^3^ *p*-value ^4^	Likelihood ratio ^4^
All GH18 genes		1		<0.001		<0.001	
	Pezizomycotina	-	-	0.007	22	0.007	22
	Emericella	-	-	0.006	14	0.006	14
	Rhizopus	-	-	0.024	1	0.024	1
	Schizosaccharomyces	-	-	0.023	3	0.023	3
Cluster A GH18 genes		1		0.002		0.002	
	Pezizomycotina	-	-	0.007	16	0.007	16
	Gibberella	-	-	0.001	5	0.006	5
	Emericella	-	-	0.004	39	<0.001	39
	Rhizopus	-	-	0.043	1	0.043	1
Cluster B GH18 genes		1		0.407		0.033	
	Hypocrea	-	-	0.002	14	<0.001	98
	Rhizopus	-	-	0.018	1	0.005	2

1 Estimates of divergence dates calibrated with *P. devonicus* as representing Sordariomycetes, giving an estimate for the fungal phylum at 1630 millions of years (Taylor and Berbee, 2006).

2 Estimates of divergence dates calibrated with *P. devonicus* as representing Pezizomycotina, giving an estimate for the fungal phylum at 923 millions of years (Taylor and Berbee, 2006).

3 Estimates of divergence dates calibrated with *P. devonicus* as representing Ascomycota, giving an estimate for the fungal phylum at 495 millions of years (Taylor and Berbee, 2006).

4 De Bie, T., Cristianini, N., Demuth, J.P. et al. 2006. CAFE: a computational tool for the study of gene family evolution. *Bioinformatics*, 22:1269–71.

**Abbreviation:** -:no significant GH18 gene family expansion or contraction.

## Figures and Tables

**Table 1:** Number of chitinase genes in different fungal species.

Species	Class	Cluster A GH18 genes	Cluster B GH18 genes	Total no. of GH18 genes
*Batrachochytrium dendrobatidis*	Chytridiomycetes	1	0	1
*Rhizopus oryzae*	Mucormycotina	9	6	15
*Schizosaccharomyces pombe*	Schizosaccharomycetes	0	1	1
*Yarrowia lipolytica*	Saccharomycetes	1	2	3
*Candida albicans*	Saccharomycetes	2	3	5
*Saccharomyces cerevisiae*	Saccharomycetes	1	1	2
*Emericella nidulans*	Eurotiomycetes	17	3	20
*Ajellomyces capsulatus*	Eurotiomycetes	7	2	9
*Uncinocarpus reesii*	Eurotiomycetes	11	3	14
*Coccidioides immitis*	Eurotiomycetes	6	3	9
*Gibberella zeae*	Sordariomycetes	16	3	19
*Hypocrea jecorina*	Sordariomycetes	11	9	20
*Magnaporthe grisea*	Sordariomycetes	11	4	15
*Neurospora crassa*	Sordariomycetes	8	4	12
*Ustilago maydis*	Ustilaginomycetes	2	1	3
*Filobasidiella neoformans*	Tremellomycetes	3	1	4
*Coprinopsis cinerea*	Agaricomycetes	7	1	8
*Phanerochaete chrysosporium*	Agaricomycetes	7	3	10

**Table 2:** Mean intraspecific levels of % amino-acid identity for GH18 subgroups in *E. nidulans* and mean interspecific levels of % amino-acid identity for GH18 subgroups between *E. nidulans* and other members of Eurotiales.

GH18 subgroup	*E. nidulans*	*A. capsulatus*	*U. reesii*	*C. immitis*
A-II	42.4	45.9	-	-
A-IV	*	52.5	56.8	57.1
A-V	58.3	56.4	52.4	54.0
C-I	22.8	12.0	15.8	19.3
C-II	25.3	-	27.1	-
B-I	*	-	35.7	35.3
B-II	*	-	29.2	30.2
B-V	*	57.5	54.6	56.2

**Abbreviations:** * Only one GH18 member in *E. nidulans*; -: no GH18 members present.

**Table 3:** Non-randomly evolving branches in the fungal GH18 gene family.

Data set	Branch ID	*p*-value^1^	Likelihood ratio^1^	Change^2^
All GH18 genes		<0.001		
	Pezizomycotina	0.005	36	7
	Coccidioides	0.032	48	−2
	Uncinocarpus	0.009	31	3
	Emericella	0.002	14	8
	Rhizopus	0.010	1	10
Cluster A GH18 genes		<0.001		
	Pezizomycotina	0.002	34	6
	Gibberella	0.026	5	5
	Coccidioides	0.002	161	−3
	Uncinocarpus	0.053	57	2
	Emericella	0.004	24	8
	Rhizopus	0.043	1	6
Cluster B GH18 genes		0.568		
	Hypocrea	0.002	14	5
	Rhizopus	0.018	1	5

1 See De Bie et al. (2006) for reference.

2 Gene family size change as compared with the most recent ancestor.
